# White Chocolate with Resistant Starch: Impact on Physical Properties, Dietary Fiber Content and Sensory Characteristics

**DOI:** 10.3390/molecules26195908

**Published:** 2021-09-29

**Authors:** Ivana Lončarević, Biljana Pajin, Jovana Petrović, Ivana Nikolić, Nikola Maravić, Đurđica Ačkar, Drago Šubarić, Danica Zarić, Borislav Miličević

**Affiliations:** 1 Faculty of Technology, University of Novi Sad, Bulevar Cara Lazara 1, 21000 Novi Sad, Serbia; ivana.loncarevic@uns.ac.rs (I.L.); biljana.pajin@uns.ac.rs (B.P.); ivananikolic@uns.ac.rs (I.N.); maravic@uns.ac.rs (N.M.); 2 Faculty of Food Technology Osijek, Josip Juraj Strossmayer University of Osijek, Franje Kuhača 18, 31000 Osijek, Croatia; dackar@ptfos.hr (Đ.A.); dsubaric@ptfos.hr (D.Š.); borislav.milicevic@ptfos.hr (B.M.); 3 Innovation Centre of the Faculty of Technology and Metallurgy Ltd., University of Belgrade, Karnegijeva 4, 11120 Belgrade, Serbia; danica.zaric@ihis-nutricionizam.rs; 4 Polytechnic in Požega, Vukovarska 17, 34000 Požega, Croatia

**Keywords:** white chocolate, resistant starch, rheology, texture, thermal properties, dietary fiber, sensory characteristics

## Abstract

Resistant starch (RS) is a part of insoluble dietary fiber, and it could be recognized as a functional food ingredient in some types of confectionery products that lack dietary fiber. Unlike dark and milk chocolate, white chocolate does not contain fat-free cocoa solids rich in dietary fiber. In the present study, 5%, 10%, and 15% of white chocolate were substituted with RS in order to improve the nutritional value of enriched white chocolate. The influence of RS on rheological, textural, and thermal properties of the chocolate fat phase was firstly investigated, and then further influence on physical properties, dietary fiber content, and sensory characteristics of enriched white chocolates were investigated. The obtained results showed that enriched chocolates had increased content of total dietary fiber and reduced total fats and protein content in accordance with the added amount of RS. At the same time, RS increased viscosity and reduced the hardness and volume mean diameter in enriched chocolates in accordance with the added amount. RS improved the nutritional composition of white chocolate by increasing the content of dietary fiber. At the same time, RS did not impair the color and sensory characteristics of enriched white chocolates.

## 1. Introduction

Modern concepts of nutrition include the great importance of functional food because functional food products, apart from their basic nutritional functions, provide many physiological benefits and reduce the risk of chronic diseases. These food products either contain or add a component with a positive health effect or eliminate a component with a negative one [1]. Dietary fibers certainly represent some of the possible functional components and among them, resistant starch is a special type of dietary fiber with functional properties [2].

Resistant starch refers to a part of starch present in the diet that escapes digestion and absorption in the small intestine and is fermented in the large intestine of humans, with the production of short–chain fatty acids (SCFA) [1,2,3]. These metabolites have important biological effects, including reduction of colon cancer precursors, systemic regulation of macronutrient metabolism, and altered secretion of hormones, which can lead to improved physical and mental health [4]. Thus, resistant starch (RS) is a form of dietary fiber and is naturally present in many starch–containing foods. The quantity can vary depending on the amount and type of present starch, processing, and storing conditions. For each person in Europe, the intake of resistant starch is approximately about 3–7 g per day [2,5,6].

Resistant starch may not be digested for several reasons, such as a compact molecular structure that limits the accessibility of digestive enzymes or the starch granules are structured in a way that prevents the digestive enzymes from breaking them down. Additionally, thermal and mechanical treatment lead to the formation of starch crystals that are resistant to enzymes digestion or selected starches that have been chemically modified by etherification, esterification, or cross–bonding, cannot be broken down by digestive enzymes [1,2].

The application of RS in food products provides many benefits, such as desirable physicochemical properties. Its functional characteristics, e.g., swelling, viscosity increase, gel formation, and water–binding capacity, ensure good handling, improves texture in the final product, as well as crispiness and expansion [2]. Resistant starch provides many technological properties, such as better appearance, texture, and mouthfeel than conventional fibers, and affecting less the sensory properties of the final products, which is very positive for consumer acceptability [1]. RS is in the form of white powder with fine particle size and along with dietary fibers fortification, it provides low caloric content of 1.6–2.8 kcal/g, especially in reduced–fat and reduced–sugar food formulations [2].

Various bioactive compounds can be used in the development of functional foods, such as soluble and insoluble fibers, prebiotics, vitamins and minerals, herbal extracts, and other phytochemicals as the main ingredients which can be used as enrichment agents for chocolate [7,8].

White chocolate is a confectionery product made of sugar, milk solids, cocoa butter, lecithin, and vanillin where the particles of sugar and milk solids are covered by a continuous fatty phase (mainly cocoa butter). Milk solids commonly consist of whole milk powder, and in some formulations skimmed milk powder, whey powder, and lactose can be used. To be named “white chocolate”, white chocolate must include whole milk powder and cocoa butter in its composition, therefore it does not contain cocoa products other than cocoa butter [9]. Therefore, white chocolate lacks valuable components such as polyphenols, minerals, and fibers [10]. Thus, the functionality of white chocolate is very low compared to dark and milk chocolate, and functional food development studies associated with white chocolate are very limited [7]. Our previous investigations were focused on the enrichment of white chocolate with polyphenol compounds by adding 6–10% of blackberry encapsulate and encapsulated green tea extract [10,11]. Barišić et al. [12] showed that cocoa shells may be used in chocolate production as a source of dietary fiber.

Taking into account all the features of resistant starch there is a very good possibility to apply RS for the development of functional white chocolate enriched with dietary fibers. Investigation of Ares et al. [13] showed that the addition of 9–15% of RS to chocolate milk desserts significantly changed sensory characteristics and significantly decreased consumers’ overall liking and acceptability. However, there are no results of testing the quality of white chocolate enriched with resistant starch in the literature. Our objective was to enrich white chocolate with dietary fiber content using resistant starch as the source of fibers. Firstly, the impact of RS on rheological, textural, and thermal properties of cocoa butter was examined with the aim to previse behavior of RS in the chocolate fat phase, and then the further impact of RS on physical and sensory properties of enriched white chocolate was analyzed.

## 2. Materials and Methods

### 2.1. Materials

The material used in this work included cocoa butter (purchased from Pionir Ltd., Subotica, Serbia), white chocolate consisted of powdered sugar, cocoa butter, milk powder, whey powder, sunflower lecithin, and vanilla aroma (produced in Pionir Ltd., Subotica, Serbia), and resistant starch ActiStarTM RT 75330 (Cargill, Inc., Wayzata, MN, USA) which is a white neutral powder with moisture content 12%, pH 5,5, according to the product specification. 

### 2.2. Plan of Experiments and Sample Preparation

In the first stage of the experiment, the model system (MS) was created with cocoa butter, which is also contained in white chocolate mass. The resistant starch (RS) was added to cocoa butter (MS0) in following amounts: 5% (MS5), 10% (MS10), and 15% (MS15). The rheological, textural, and thermal properties of the model system were examined.

#### 2.2.1. Sample Preparation and Pre-Crystallization of Model System 

The pre-crystallization of cocoa butter and the mixture of cocoa butter and resistant starch was carried out in a modified Brabender farinograph where the original kneader is connected with two thermostats (Lauda Ecoline Staredtition E 215 T, Lauda-Königshofen, Germany) with two-way taps. 100 g of chopped cocoa butter was placed in a farinograph kneader to melt at 50 °C for 45 min. Then the stirrer of the farinograph was switched on and the cocoa butter was stirred at 50 °C for 15 min, followed by another 10 min at 25 °C and another 15 min at 32 °C. The pre-crystallized cocoa butter was then poured into 10 × 10 g plastic molds and cooled in a refrigerator at 5 °C for 60 min. The same procedure was carried out in order to produce the sample MS0 and model system samples (MS5, MS10, and MS15), where a certain amount of resistant starch was added to melted cocoa butter after 45 min at 50 °C. 

In the second stage of the experiment, the influence of RS on the quality of white chocolate was observed. The examined samples were: white chocolate without RS (WC0) and enriched white chocolate with 5% (WC5), 10% (WC10), and 15% (WC15) of RS.

#### 2.2.2. Sample Preparation and Pre-Crystallization of Chocolate Mass

The pre-crystallization of white chocolate mass was also carried out in a modified Brabender farinograph where 100 g of chopped chocolate was placed in a farinograph kneader to melt at 40 °C for 45 min. Then the stirrer of the farinograph was switched on and the chocolate mass was stirred at 40 °C for 15 min, followed by another 10 min at 26 °C and another 15 min at 29 °C. The pre-crystallized chocolate mass was then poured into 10 × 10 g plastic molds and cooled in a refrigerator at 5 °C for 60 min. The same procedure was carried out in order to pre-crystallize the control sample of chocolate (WC0) and enriched white chocolate (WC5, WC10, and WC15), where certain concentrations of resistant starch were added to melted cocoa chocolate mass after 45 min at 40 °C.

### 2.3. Methods

#### 2.3.1. Chemical Composition of Chocolate Samples

Moisture, protein, fat, and ash contents of chocolate samples were determined after one day of storage, according to the methods described in the AOAC (No. 931.04, No. 939.02, No. 963.15, and No. 930.22, respectively) [14]. The total dietary fiber content of resistant starch and chocolate samples is determined by method No. 958.23. Total non-fiber carbohydrate contents are determined by difference (100—% moisture, % protein, % fat, % ash, and % fiber content). 

#### 2.3.2. Rheological Properties of Model System and Chocolate Mass

The rheological properties of samples were determined by a rotational rheometer Rheo Stress 600 (Haake, Germany) at the temperature 40 ± 1 °C [15]. The flow curves were determined by applying the method of the hysteresis loop using a concentric cylinder system (sensor Z20 DIN). Shear rate was increased from 0 s^−1^ to 60 s^−1^ during a period of 180 s and then was kept constant for 60 s at max. speed of 60 s^−1^ and after that was reduced from 60 s^−1^ to 0 s^−1^, within 180 s. 

#### 2.3.3. Textural Properties of Model System and Chocolate

The determination of textural properties of the model system and chocolate was performed using Texture Analyser (Stable Micro System, UK) following the 3-Point Bending Rig HDP/3PB method. 

#### 2.3.4. Thermal Properties of Model System and Chocolate

Differential scanning calorimetry DSC 910, Thermal analyzer 990, and Dynamic mechanical analyzer (Du Point Instruments, USA) were used to determine the thermal profile of the model system and chocolate according to Lončarević et al. [16]. 

#### 2.3.5. Particle Size Distribution of Resistant Starch and Chocolate

Particle size distribution of resistant starch and chocolate samples were determined using the Mastersizer 2000 laser diffraction particle size analyzer (Malvern Instruments, England). The Scirocco dispersion unit was used for dispersing resistant starch in the air, while the Hydro 2000 μP dispersion unit was used for dispersing chocolate in sunflower oil. The samples were added at ambient temperature until an adequate obscuration was obtained (5–10% for dry samples and 10–20% for liquid samples). The results were quantified as the volume-based particle size distribution by means of the Mastersizer 2000 software.

#### 2.3.6. Color of Model System and Chocolate

The surface color of resistant starch, model system, and chocolate samples were determined by MINOLTA Chroma Meter CR-400 (Minolta Co., Ltd., Osaka, Japan) using D 65 lighting, a 2° standard observer angle, and an 8-mm aperture in the measuring head. The obtained results were expressed in terms of: L*—lightness, a*—redness to greenness and b*—yellowness to blueness. The CIE color difference was also calculated using the formula is defined as:

∆E = [ (L* − L_0_*)^2^ + (a* − a_0_*)^2^ + (b* − b_0_*)^2^]^1/2^ (1)

where L_0_*, a_0_*, and b_0_* are values for the control white chocolate sample [17].

#### 2.3.7. Sensory Analyses

The sensory panel was comprised of eight assessors (five female and three male), food technologists. They had been selected according to the guidelines of the ISO 8586:2012 standard [18]. The attribute list was obtained through discussion with the panel leader. Five white chocolate samples representing a wide range of sensory characteristics were presented to the assessors. Through a consensus process, they agreed on the most adequate attributes to fully describe the dynamics of the sensory characteristics of samples. Next, sample evaluation cards with the panelist list of definitions of attributes and their lowest and highest intensity were prepared. 

The final list of attributes included 10 attributes covering appearance, aroma, flavor, and texture to be used in the examination and the panelists were asked to rate the intensity of each attribute using a 7-point scale [19]: color uniformity (1—non uniform to 7—uniform); glow (1—matte to 7—extremely glossy); surface damage (1—none to 7—many); hardness—the easiness with which samples could be broken into two parts (1—extremely soft to 7—extremely hard); smoothness—the proportion of small solids between teeth during chewing (1—smooth to 7 - grainy); melting—the time it takes for pieces of chocolate to transform into a liquid in the mouth (1—slow to 7—quick); cocoa flavor—characteristic taste related to the presence of cocoa (1—poorly expressed to 7—very expressed); milk powder flavor—characteristic taste related to the presence of milk powder (1—poorly expressed to 7—very expressed); sweetness—characteristic taste related to the presence of sucrose (1—poorly expressed to 7—very expressed); floury—sensation of flour in the mouth (1—non-existent to 7—very expressed). 

The tests were conducted in the sensory laboratory at the Faculty of Technology Novi Sad, in individual cabinets illuminated with white light, designed in accordance with ISO 8589 [20]. The chocolate pieces were unwrapped and served at room temperature (21 °C) on three-digit numbered plastic plates. Mineral water at room temperature and diced peeled apple were served between sample servings. The average point number was calculated for each sample.

#### 2.3.8. Statistical Analysis

All experiments were performed in triplicate (except the sensory analysis that included eight panelists). The obtained results were statistically tested using the ANOVA method and the means were compared by one-factor analysis at variance with subsequent comparisons by Duncan’s test at a significance level at 0.05 using software Statistica 13.3 Software (TIBCO Software Inc., Paolo Alto, CA, USA, 2016).

## 3. Results and Discussion

### 3.1. The Impact of RS on Rheological and Textural Properties of Model System and Chocolate

**Figure 1:**
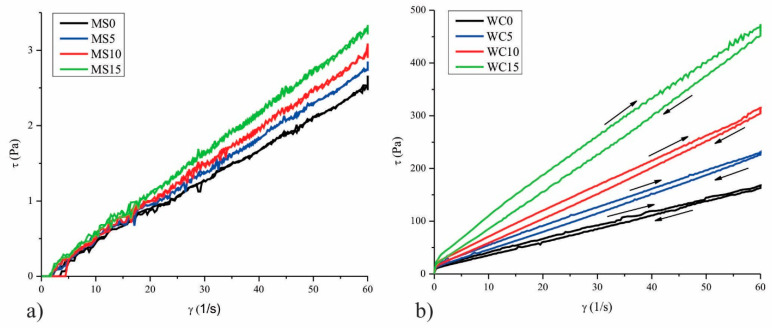
Flow curves of (**a**) model system and (**b**) white chocolate with different amounts of RS.

Cocoa butter represents the main part of the continuous phase in chocolate, and thus it is responsible for the dispersion of all other constituents and for the physical behavior of chocolate [21]. Rheological measurements of confectionery fat-based systems predict interactions of components within the system and behavior during processing [22]. In new product development, it is necessary to control the rheological properties of chocolate mass due to their reflection on the quality of the final product [23]. The impact of 5, 10, and 15% of RS on the rheological properties of cocoa butter and white chocolate is shown in Figure 1.

The viscosity of the model system on maximum shear rate (60 1/s) increases with an increase in the amount of RS. White chocolate samples exhibit a thixotropic type of flow. The addition of RS caused the reduction of the white chocolate fat phase consisted of cocoa butter and milk fat and thus increased viscosity of white chocolate mass in accordance with the added amount. The obtained flow curves of white chocolate were fitted using the Casson model to get the following parameters: Cason yield stress (Pa) and Casson viscosity (Pa·s). The viscosity of the model system and rheological parameters of white chocolate are presented in Table 1. 

Viscosity at the maximum share rate of the model system and Casson viscosity of white chocolate increased with increasing RS from 5 to 15% where all values differ significantly from each other (*p* ˂ 0.05). Increased values of these rheological parameters for model systems and white chocolate samples indicated reduced flow properties of observed systems. 

Furthermore, the addition of all amounts of RS to white chocolate increased the values of Casson yield stress, where all values differ significantly (*p* ˂ 0.05) from the control sample of white chocolate (WC0). Yield stress relates to the energy required to initiate chocolate flow and is important in keeping solid particles in suspension and in the coating of solid surfaces. Fat fills spaces between particles in molten chocolate and reduces resistance to flow [24], thus reduced-fat amount by the addition of RS caused higher particle interaction and increases the rheological parameters. The increasing of added RS reduced the fat phase amount in enriched white chocolate and comparing to the control sample (WC0), there was a less continuous fat phase for coating the solid particles, which makes the initial flow more difficult. 

The addition of 5–15% of RS to cocoa butter significantly decreased (*p* ˂ 0.05) the hardness values of model system samples compared to MS0. However, the values of MS10 and MS15 do not differ significantly between each other. 

Similarly, the decrease in the amount of fat phase in enriched chocolates caused by the addition of RS led to a statistically insignificant decrease (*p* > 0.05) of hardness value in the sample WC5 compared to the control sample WC0 and to a significant decrease (*p* ˂ 0.05) of hardness in samples WC10 and WC15 compared to WC0. Generally, the addition of higher amounts of RS (10 and 15%) contributed to a reduced hardness of chocolate, since the amount of cocoa butter decreased. Various studies have been carried out on the relationship between the composition of chocolate and its hardness, in terms of correlation between the hardness of chocolate and fat levels [25]. 

### 3.2. The Impact of RS on Thermal Properties of Model System and Chocolate

DSC parameters—onset temperature (T_onset_), peak temperature (T_peak_), conclusion temperature (T_end_), and enthalpy (J/g) are presented in Table 2.

T_onset_ corresponds to the temperature at which a specific crystal form starts to melt. T_peak_ presents the maximum temperature at which the melting rate is the greatest. T_end_ is the end temperature of melting [24]. 

The addition of RS to cocoa butter did not significantly affect the T_onset_ values of the model system. T_peak_ values of the model system decreased with increasing the amount of RS. The addition of 5% of RS to the model system did not significantly decrease the T_peak_ value of MS5 and significantly (*p* ˂ 0.05) decreased the values of MS10 and MS15. However, there is no significant difference between T_peak_ values of MS samples with all amounts of RS added. The values of T_end_ of the model system also decreased with increasing the amount of RS. Only the maximum amount of RS significantly (*p* ˂ 0.05) decreased T_end_ value comparing to the control sample of the model system. The control sample MS0 has significantly (*p* ˂ 0.05) the highest value of enthalpy. 

All white chocolate samples were in the temperature range expected for chocolate melting profiles. All DSC parameters are lower in white chocolate compared to the model system. Enthalpy values also significantly (*p* ˂ 0.05) decreased in enriched chocolates compared to white chocolate with no RS added in accordance with the increase of RS amount. On the other hand, there is no significant difference between T_end_ values of all chocolate samples. Contrary to the model system, T_onset_ and T_peak_ values significantly (*p* ˂ 0.05) increased in sample WC15, while the values of the other chocolate samples do not differ statistically between each other.

### 3.3. The Impact of RS on Particle Size Distribution of Chocolate

**Figure 2:**
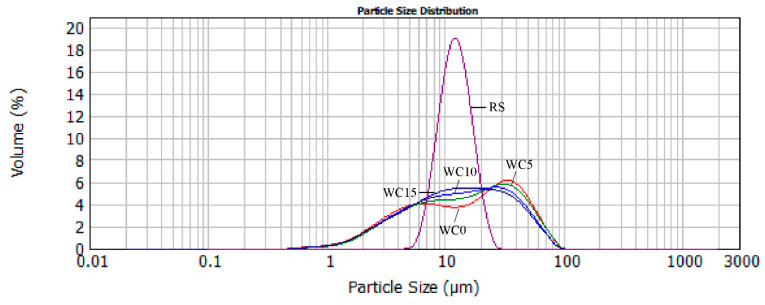
Particle size distribution of RS, white chocolate, and enriched chocolate samples.

Particle size distribution influences chocolate structure–inter-particle interactions and network microstructure, rheology, and texture. Specific surface area and mean particle size influence yield stress, plastic viscosity, and hardness [26]. Figure 2 represents the particle size distribution of RS, white chocolate, and enriched white chocolate samples. The particle size distribution of white chocolate is multimodal. In general, RS has smaller particle sizes compared to white chocolate, however, it can be noticed that the largest volume of RS consists of particles with diameters in intervals of 10–20 μm, which suggests that RS is a suitable ingredient for addition in the chocolate formulation. The higher amount of added RS (from 5 to 15%) in white chocolate increased the volume of chocolate containing the particles with diameter 10–20 μm as can be seen from Figure 2. 

The parameters of the particle size distribution of RS and chocolate samples are presented in Table 3.

The addition of all amounts of RS to white chocolate does not significantly affect the d(0.1) parameter in enriched chocolates and significantly (*p* ˂ 0.05) affects all other particle size parameters compared to the control sample of white chocolate. The values of parameters d(0.5), d(0.9), and D[4,3] decrease with increasing the amount of RS. However, there is no significant difference between samples WC0 and WC5 and samples WC10 and WC15. The volume mean diameter D[4,3] decreases from 21.32 µm in WC0 to 18.82 µm in WC15 which is in accordance with the fact that particles in chocolate are desirable to be in the interval 15–30 μm [27].

### 3.4. The Photographs and Surface Color of White Chocolate

**Figure 3:**
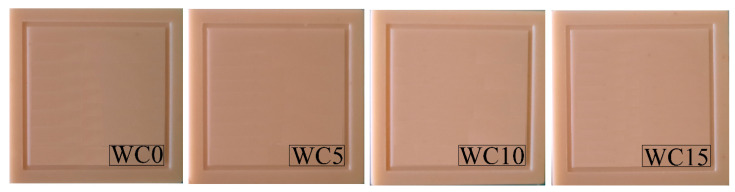
Photograph of white chocolate and white chocolates with RS.

The photograph of chocolate samples, indicating the impact of added RS on their appearance, is presented in Figure 3.

The addition of 0–15% RS to white chocolate did not impair the appearance and surface color of white chocolate, although many functional ingredients cause a color change in chocolate products [28]. The values of L* (lightness), a* (red tone), and b* (yellow tone) measured on the surface of RS and their further influence on the surface color of enriched chocolate samples are presented in Table 4.

RS has the significantly (*p* < 0.05) highest L* value and significantly (*p* < 0.05) lowest a* and b* value indicating almost white surface color with a small share of a red and yellow tone. However, the addition of all amounts of RS to white chocolate did not significantly affect the lightness of the enriched chocolate surface. On the other hand, all enriched chocolate samples have significantly (*p* < 0.05) lower values of a* compared to the control sample of white chocolate. Furthermore, the addition of 5–15% of RS generally caused the decrease in b* values of enriched chocolates, where WC10 and WC15 have significantly (*p* < 0.05) lower values compared to WC0. Thus, the addition of RS in the white chocolate structure reduced the amount of red and yellow tone in observed white chocolate samples compared to the control one, according to the instrumental color determination.

The average values of parameter ∆E were 0.49, 0.55, and 0.96 respectively. Obtained results indicate that the color difference between each sample and WC0 is very low. When the values of ∆E are between 0.2 and 0.1, it means that there is very little visibility of the color difference between samples registered by the human eye [29].

### 3.5. The Impact of RS on Chemical Composition of Chocolate Samples

Mean values for the chemical composition of chocolate samples are presented in Table 5. The addition of RS affects the chemical properties of chocolate, as significant differences (*p* < 0.05) were obtained among all samples compared to the control sample, observing all chemical characteristics. Samples with starch addition had significantly higher moisture content compared to the control sample. In relation to the fat and protein parameters all samples were statistically different (*p* < 0.05) among them. As starch does not contain high amounts of fat and protein, it was expected that the amount of these parameters would decrease evenly with increasing starch content in the chocolate samples.

As white chocolate contains a negligible proportion of fiber, the primary goal of our research was to increase fiber content in this product. Resistant starch contains 86.45% of fiber (the result is not presented in Table 5), so the addition of RS led to a significant increase in the content of dietary fiber in white chocolate. With the addition of 15% of RS, the fiber content increased to as much as 9%. Pursuant to the Serbian [30], European [31], and American [32] regulations, sample WC5 can carry nutritional claims on the label of products as “fiber source” (content 3.0 g fiber/100 g of solid food) and samples WC10 and WC15 as “high fiber sources” (content 6.0 g fiber/100 g solid food).

### 3.6. The Impact of RS on Sensory Characteristics of White Chocolate

The attributes of surface appearance (color uniformity, glow, and surface damage) did not differ significantly (*p* > 0.05) among the chocolate samples (Table 6). All samples had a perfect surface, without damage, good gloss, with clear engraving, and without separating white spots, as can be seen in Figure 3.

The control sample (WC0) presented the highest mean value for hardness and differed significantly (*p* < 0.05) from the other chocolate samples. With the increase in the proportion of resistant starch in the chocolate samples, there was a decrease in hardness. Sample WC15 had the lowest value for hardness, significantly lower than other samples, while samples WC5 and WC10 did not differ significantly in hardness, according to the panelists. These results are in accordance with the instrumental hardness measurement presented in Table 1.

The resistant starch had a statistically significant influence on the chocolate smoothness only in the case of the addition of the largest quantity (15%), while it did not have a significant influence on the chocolate melting in the mouth.

The pleasant flavor and taste are the most important sensory attributes of chocolate because they determine the acceptance by consumers [33]. As RS has a neutral taste, its addition did not give rise to a foreign taste, except that it affected the reduction of cocoa flavor, milk powder flavor, and sweetness intensity. The evaluators noticed only a mild floury taste which is a consequence of the increased proportion of fine particles in the chocolate samples and the decrease in the proportion of fatty phase necessary to cover all powder particles.

Ares et al. [13] mentioned that the resistant starch addition up to 15% caused significant changes in the sensory characteristics of the chocolate milk desserts and a significant decrease in consumers’ overall liking. However, this was not the case with the addition of resistant starch into white chocolate.

Observing the results of sensory analysis, it can be said that the addition of resistant starch to white chocolate up to 15% did not affect the deterioration of sensory characteristics. The differences in the sensory attributes between samples, noticed by trained panelists are very small so that consumers probably would not even be able to notice them.

## 4. Conclusions

The effects of resistant starch introduction to the formulation of white chocolate samples were investigated in terms of physical properties, nutritional value, and sensory characteristics. Resistant starch addition increased the water and dietary fiber content of white chocolate samples. The rheological properties of white chocolate samples showed strong dependence on the resistant starch addition to the formulation. Viscosity values of tested samples significantly increased with the increasing amount of resistant starch in the sample formulation. However, resistant starch supplemented chocolates had similar sensory characteristics as the control samples.

## Figures and Tables

**Table 1:** Rheological parameters and hardness of model system and chocolate.

Model System (MS)			White Chocolate (WC)			
Sample	Viscosity at Max Shear Rate (Pa·s)	Hardness (kg)	Sample	Casson Yield Stress (Pa)	Casson Viscosity (Pa·s)	Hardness (g)
MS0	0.042 ± 0.0010 ^a^	4.98 ± 0.38 ^c^	WC0	1.78 ± 0.23 ^a^	2.25 ± 0.10 ^a^	3.07 ± 0.11 ^b^
MS5	0.046 ± 0.0006 ^b^	4.17 ± 0.15 ^b^	WC5	2.04 ± 0.45 ^b^	3.11 ± 0.21 ^b^	2.93 ± 0.30 ^b^
MS10	0.050 ± 0.0016 ^c^	3.38 ± 0.13 ^a^	WC10	2.35 ± 0.22 ^bc^	4.47 ± 0.23 ^c^	2.22 ± 0.20 ^a^
MS15	0.055 ± 0.0010 ^d^	3.48 ± 0.28 ^a^	WC15	2.86 ± 0.15 ^c^	6.51 ± 0.12 ^d^	1.74 ± 0.23 ^a^

Values represent the mean value of 3 measurements. Values followed by the same letter within the same column are not significantly different (*p* > 0.05) according to Duncan’s test.

**Table 2:** Thermal parameters of model system and of chocolate samples.

Sample	T_onset_ (°C)	T_peak_ (°C)	T_end_ (°C)	Enthalpy (J/g)
Model System (MS)				
MS0	30.99 ± 0.43 ^a^	34.41 ± 0.29 ^b^	39.93 ± 0.30 ^b^	135.83 ± 5.79 ^b^
MS5	30.18 ± 0.66 ^a^	33.92 ± 0.23 ^ab^	39.48 ± 0.45 ^b^	121.03 ± 3.33 ^a^
MS10	30.57 ± 0.85 ^a^	33.88 ± 0.16 ^a^	39.14 ± 0.35 ^ab^	120.20 ± 2.30 ^a^
MS15	30.67 ± 1.03 ^a^	33.87 ± 0.34 ^a^	38.48 ± 0.45 ^a^	124.43 ± 3.48 ^a^
White Chocolate (WC)				
WC0	27.07 ± 0.07 ^a^	30.82 ± 0.10 ^a^	35.88 ± 0.34 ^a^	24.81 ± 0.17 ^c^
WC5	26.64 ± 0.21 ^a^	30.60 ± 0.09 ^a^	35.60 ± 0.36 ^a^	23.10 ± 0.12 ^b^
WC10	27.13 ± 0.11 ^a^	30.74 ± 0.14 ^a^	35.91 ± 0.21 ^a^	23.26 ± 0.15 ^b^
WC15	27.72 ± 0.19 ^b^	31.21 ± 0.29 ^b^	35.74 ± 0.22 ^a^	18.87 ± 0.12 ^a^

Values represent the mean value of 3 measurements. Values followed by the same letter within the same column (within the values for model system or white chocolate) are not significantly different (*p* > 0.05) according to Duncan’s test.

**Table 3:** Particle size parameters of RS and enriched chocolate.

Sample	Particle Size Parameters (µm)			
	d(0.1)	d(0.5)	d(0.9)	D[4,3]
RS	8.09 ± 0.19 ^b^	12.05 ± 0.13 ^a^	17.86 ± 0.22 ^a^	12.60 ± 0.25 ^a^
WC0	3.03 ± 0.13 ^a^	15.48 ± 0.78 ^c^	48.67 ± 0.71 ^c^	21.32 ± 0.53 ^c^
WC5	3.21 ± 0.18 ^a^	15.24 ± 0.22 ^c^	48.10 ± 1.36 ^c^	21.13 ± 0.48 ^c^
WC10	3.16 ± 0.04 ^a^	13.67 ± 0.18 ^b^	44.29 ± 0.58 ^b^	19.34 ± 0.24 ^b^
WC15	3.19 ± 0.04 ^a^	13.21 ± 0.08 ^b^	43.22 ± 0.69 ^b^	18.82 ± 0.20 ^b^

Values represent the mean value of 3 measurements. Values followed by the same letter within the same column are not significantly different (*p* > 0.05) according to Duncan’s test.

**Table 4:** Color (CIE L*a*b* system) on the surface of RS and enriched white chocolate samples.

Sample	CIE L*a*b* System			
	L*	a*	b*	∆E
RS	98.12 ± 0.76 ^b^	1.53 ± 0.06 ^a^	1.04 ± 0.02 ^a^	-
WC0	73.18 ± 0.17 ^a^	2.06 ± 0.03 ^d^	19.23 ± 0.33 ^c^	-
WC5	73.51 ± 0.97 ^a^	1.96 ± 0.04 ^c^	18.88 ± 0.19 ^bc^	0.49 ± 0.03 ^a^
WC10	73.34 ± 0.48 ^a^	1.91 ± 0.04 ^bc^	18.73 ± 0.45 ^b^	0.55 ± 0.06 ^a^
WC15	74.01 ± 1.06 ^a^	1.87 ± 0.08 ^b^	18.78 ± 0.40 ^b^	0.96 ± 0.07 ^b^

L*a* b* values represent the mean value of 3 measurements. Same letter in the superscript (^a–c^) within the same column indicates values are not significantly different (*p* > 0.05) according to Duncan’s test.

**Table 5:** Chemical composition of chocolate samples.

	Moisture	Fat	Protein	Ash	Fiber	Carbohydrates
Sample			(%)			
WC0	0.71 ± 0.02 ^a^	32.45 ± 0.06 ^a^	5.28 ± 0.06 ^a^	1.16 ± 0.06 ^a^	0.35 ± 0.06 ^a^	60.01 ± 1.36 ^a^
WC5	1.39 ± 0.44 ^b^	29.91 ± 0.21 ^b^	5.06 ± 0.07 ^b^	1.14 ± 0.06 ^b^	3.50 ± 0.28 ^b^	59.00 ± 0.80 ^b^
WC10	1.46 ± 0.04 ^b^	26.59 ± 0.24 ^c^	4.70 ± 0.07 ^c^	1.11 ± 0.22 ^c^	7.11 ± 0.14 ^c^	59.03 ± 0.11 ^b^
WC15	1.81 ± 0.02 ^b^	23.79 ± 0.36 ^d^	4.44 ± 0.09 ^d^	1.15 ± 0.11 ^d^	9.76 ± 0.48 ^d^	59.05 ± 0.01 ^b^

Values represent the mean value of 3 measurements. Values followed by the same letter within the same column are not significantly different (*p* > 0.05) according to Duncan’s test.

**Table 6:** Sensory characteristics of chocolate samples.

Sample				
	WC0	WC5	WC10	WC15
Color uniformity	6.94 ± 0.09 ^a^	6.90 ± 0.14 ^a^	6.92 ± 0.13 ^a^	6.84 ± 0.21 ^a^
Glow	3.62 ± 0.13 ^a^	3.54 ± 0.15 ^a^	3.52 ± 0.11 ^a^	3.50 ± 0.16 ^a^
Surface damage	1.08 ± 0.11 ^a^	1.10 ± 0.14 ^a^	1.10 ± 0.14 ^a^	1.12 ± 0.13 ^a^
Hardness	4.52 ± 0.15 ^a^	4.20 ± 0.07 ^b^	4.08 ± 0.13 ^b^	3.86 ± 0.05 ^c^
Smoothness	1.0 ± 0.00 ^a^	1.06 ± 0.08 ^a,b^	1.16 ± 0.11 ^a,b^	1.18 ± 0.18 ^b^
Melting	5.88 ± 0.26 ^a^	5.74 ± 0.32 ^a^	5.62 ± 0.34 ^a^	5.60 ± 0.33 ^a^
Cocoa flavor	4.26 ± 0.27 ^a^	4.04 ± 0.11 ^b^	3.68 ± 0.18 ^c^	3.36 ± 0.19 ^c^
Milk powder flavor	5.06 ± 0.09 ^a^	4.80 ± 0.16 ^b^	4.70 ± 0.15 ^b^	4.34 ± 0.21 ^c^
Sweetness	5.44 ± 0.15 ^a^	5.40 ± 0.16 ^a^	5.14 ± 0.11 ^b^	5.06 ± 0.09 ^b^
Floury taste	1.00 ± 0.00 ^a^	2.32 ± 0.22 ^b^	2.40 ± 0.16 ^b^	2.42 ± 0.13 ^b^

Values followed by the same letter within the same column are not significantly different (*p* > 0.05) according to Duncan’s test.

## Data Availability

Not applicable.

## Sample Availability

Samples of the compounds are available from the authors.
